# Increase in candidemia cases and emergence of fluconazole-resistant *Candida parapsilosis* and *C. auris* isolates in a tertiary care academic hospital during the COVID-19 pandemic, Greece, 2020 to 2023

**DOI:** 10.2807/1560-7917.ES.2024.29.29.2300661

**Published:** 2024-07-18

**Authors:** Maria Siopi, Panagiota-Christina Georgiou, Paschalis Paranos, Maria-Ioanna Beredaki, Aikaterini Tarpatzi, Eleni Kalogeropoulou, Sofia Damianidou, Alexandra Vasilakopoulou, Polyxeni Karakosta, Spyros Pournaras, Joseph Meletiadis

**Affiliations:** 1 Clinical Microbiology Laboratory, “Attikon” University General Hospital, Medical School, National and Kapodistrian University of Athens, Athens, Greece

**Keywords:** candidaemia, *C. auris*, fluconazole-resistant *C. parapsilosis*, epidemiology, Greece, healthcare-associated infections, fungal infections, antimicrobial resistance, infection control, laboratory surveillance, laboratory

## Abstract

**Background:**

The COVID-19 pandemic and the emergence of *Candida auris* have changed the epidemiological landscape of candidaemia worldwide.

**Aim:**

We compared the epidemiological trends of candidaemia in a Greek tertiary academic hospital before (2009–2018) and during the early COVID-19 (2020–2021) and late COVID-19/early post-pandemic (2022–2023) era.

**Methods:**

Incidence rates, species distribution, antifungal susceptibility profile and antifungal consumption were recorded, and one-way ANOVA or Fisher’s exact test performed. Species were identified by MALDI-ToF MS, and in vitro susceptibility determined with CLSI M27-Ed4 for *C. auris* and the EUCAST-E.DEF 7.3.2 for other *Candida* spp.

**Results:**

In total, 370 candidaemia episodes were recorded during the COVID-19 pandemic. Infection incidence (2.0 episodes/10,000 hospital bed days before, 3.9 during the early and 5.1 during the late COVID-19 era, p < 0.0001), *C. auris* (0%, 9% and 33%, p < 0.0001) and fluconazole-resistant *C. parapsilosis* species complex (SC) (20%, 24% and 33%, p = 0.06) infections increased over time, with the latter not associated with increase in fluconazole/voriconazole consumption. A significant increase over time was observed in fluconazole-resistant isolates regardless of species (8%, 17% and 41%, p < 0.0001). Resistance to amphotericin B or echinocandins was not recorded, with the exception of a single pan-echinocandin-resistant *C. auris* strain.

**Conclusion:**

Candidaemia incidence nearly tripled during the COVID-19 era, with *C. auris* among the major causative agents and increasing fluconazole resistance in *C. parapsilosis* SC. Almost half of *Candida* isolates were fluconazole-resistant, underscoring the need for increased awareness and strict implementation of infection control measures.

**Key public health message**

**What did you want to address in this study and why?**

The impact of COVID-19 on the epidemiology of candidaemia has not been fully explored. We therefore studied the epidemiology of candidaemia in a tertiary care university hospital during the COVID-19 pandemic and compared with the pre-COVID-19 era.

**What have we learnt from this study?**

The cases of candidaemia nearly tripled during the COVID-19 era. *C. auris* was the most common and together with *C. parapsilosis* SC accounted for two thirds of *Candida* bloodstream infections, leaving *C. albicans* as the third most common pathogen after the COVID-19 pandemic. Fluconazole resistance in *C. parapsilosis* SC increased. Almost half of all *Candida* isolates were resistant to fluconazole.

**What are the implications of your findings for public health?**

Changes in epidemiology and more difficult to treat fungal infections require surveillance and prompt treatment to contain the spread of resistant *Candida* isolates.

## Introduction

Since the onset of the COVID-19 pandemic, a growing number of studies have indicated a significant rise in the number of secondary invasive fungal diseases, including candidaemia [1]. In fact, the global spread of the COVID-19 outbreak posed serious healthcare challenges, comprising staff and equipment shortages, as well as collateral damage to infection control measures [2]. More recently, the exponential increase and geographical spread of *Candida auris*-related invasive infections have raised concerns [3,4]. The epidemiology of *C. auris* bloodstream infections (BSI) in Greece has been described only in a limited number of cases [4,5], mainly in COVID-19 critically ill patients [6-8], which may not be indicative of the extent of their occurrence in the general patient population of Greece.

Meanwhile, although guidelines exist to guide therapeutic decisions [9,10], management of candidaemic patients may require a tailored approach owing to the considerable geographical variation in the distribution of the causative pathogens and their corresponding resistance patterns [11]. Hence, empirical antifungal strategies must be guided by local epidemiology, taking into account the local ecology as determined through diligent surveillance with provision of feedback at regular intervals [12]. To date, the contemporary epidemiology of candidaemia in Greece remains unknown since the little data available focused mostly on intensive care unit (ICU)-acquired infections [7,8,13,14].

In March 2020, Attikon University General Hospital was appointed as one of the referral centres providing COVID-19 care services in Attica, which is the most densely populated region of Greece and encompasses the entire metropolitan area of Athens. During the first 2 years of the pandemic, several changes in Attikon’s routine workflow were implemented in order to accommodate the surge of COVID-19 patients. These included the cancellation of non-urgent surgical and medical procedures as well as outpatient visits, emergency conversion of one of the two existing ICUs to an exclusively COVID-19 ICU and the creation of a new ICU, and transformation of several wards to specialised COVID-19 non-critical care units that were functioning for various time periods depending on the gravity of the transmission waves of COVID-19 in Greece. Of note, Attikon is a tertiary academic hospital that attends cases of high complexity, including adult, paediatric and neonatal ICUs, haematology and oncology wards as well as bone marrow transplantation and HIV/AIDS units, whose services could not be completely interrupted, but were diminished to the least possible degree.

We performed a systematic review of the existing literature related to candidaemia in Greece during the COVID-19 pandemic and investigated the epidemiology of the infection before [15] and during the pandemic up to December 2023 in Attikon to provide an overview of the epidemiological situation and the impact of the pandemic.

## Methods

### Literature review

We carried out electronic searches in the PubMed, Google Scholar and Web of Science databases using the keywords ‘*Candida*’, ‘fungaemia’, ‘candidaemia’ and ‘bloodstream infection’ in conjunction with ‘Greece’ and/or ‘Greek’ in February 2024. Additional handpicked searches in the bibliographies of the articles retrieved were also performed. Only studies written in English and conducted during the COVID-19 era (1 January 2020 to 31 December 2023), were considered. From the studies retrieved, we extracted data regarding the geographic region, observation time period, study design, patient population, number of cases, candidaemia incidence rates, the relative proportions of *Candida* spp. and the antifungal susceptibility profiles of isolates (including the methodology and interpretation criteria used).

### Study period, setting and population

All microbiologically confirmed candidaemia cases in adult patients hospitalised at Attikon between (i) 2009 and 2018 (pre-COVID-19 era) [15], (ii) 1 January 2020 and 31 December 2021 (early COVID-19 era), and (iii) 1 January 2022 and 31 December 2023 (late COVID-19/early post-pandemic era, hereafter referred to as late COVID-19 era), were retrospectively analysed. Attikon is a modern teaching hospital and the largest in the West Attica region (western part of the agglomeration of Athens), with a potential of 750 beds and an average of 52,220 admissions per year in the pre-COVID-19 era [15]. It serves West and South Attica as well as most Aegean islands (second regional health authority of a population of ca. 2 million people).

Candidaemia was defined as the recovery of *Candida* spp. from at least one blood culture obtained during hospitalisation. Subsequent positive blood cultures with the same *Candida* spp. from a single patient were considered a new episode if the episodes occurred more than 4 weeks apart, along with the clearance of the prior blood culture (negative blood culture 14 days after the first negative blood culture) and resolution of all clinical features of the infection. Blood cultures yielding different *Candida* spp., independently of the time interval between the new and the prior positive blood culture, were considered to represent new episodes.

Positive blood cultures were inoculated onto blood agar, MacConkey agar and chocolate agar incubated at 37 °C, and Sabouraud glucose agar with gentamicin and chloramphenicol and chromogenic Brilliance *Candida* agar incubated at 30 °C, for up to 48 hours (all agar plates from Oxoid, Athens, Greece). Patients with mixed candidaemia, identified as the isolation of ≥ 2 different *Candida* spp. from a single blood culture sample, were included.

COVID-19 patients were those who tested positive for severe acute respiratory syndrome coronavirus 2 (SARS-CoV-2) RNA in respiratory specimens (either on admission or later in their hospital course before the occurrence of candidaemia) using commercial real-time RT-PCR assays available at the time. The group of non-COVID-19 patients, those who had no evidence of COVID-19 diagnosed at any time point throughout the study period, encompassed those with *Candida* BSI.

### Species identification and antifungal susceptibility testing

*Candida* BSI isolates were identified to species level by MALDI-ToF MS (Bruker Daltonics, Bremen, Germany). *C. auris* in vitro susceptibility was assessed using the Clinical and Laboratory Standards Institute (CLSI) M27-Ed4 [16] with the Centers for Disease Control and Prevention’s (CDC) tentative resistance breakpoints [17] and previously proposed CLSI epidemiological cut-off values [18]. Hence, *C. auris* isolates were classified as non-resistant/resistant to amphotericin B, echinocandins and fluconazole and wild type/non-wild type to the other drugs. For other *Candida* spp., the in vitro susceptibility profile was determined with the European Committee on Antimicrobial Susceptibility Testing (EUCAST) Definitive Document (E.DEF) 7.3.2 [19] following the corresponding guidelines for interpreting the minimum inhibitory concentration (MIC).

### Statistical analysis

The incidence of candidaemia was expressed as the ratio of *Candida* BSI episodes per 1,000 hospital and unit (ICU and non-ICU) admissions and per 10,000 hospital and unit (ICU and non-ICU) bed days. Antifungal consumption data were reported as defined daily doses (DDD) and expressed in DDD/100 hospital bed days using the antimicrobial consumption (AMC) tool 2019 version 1.9.0 (World Health Organization (WHO) Collaborating Centre for Drug Statistics Methodology, Oslo, Norway). Trend over time was evaluated by one-way ANOVA followed by post-test for linear trend. Changes in species distribution and in vitro susceptibility over the years were assessed statistically using Fisher’s exact test. A p value of < 0.05 was considered to reveal a statistically significant difference. All data were analysed using the statistics software package GraphPad Prism 8.0 (GraphPad Software, San Diego, United States (US)).

## Results

### Literature review

We retrieved seven articles whose extracted data are presented in Table 1. Overall, the study periods encompassed the early COVID-19 era (March 2020 to December 2021, 6/7 articles), while all articles reported data from single hospitals. The majority of the studies (4/7) were performed in ICUs (3/7 in COVID-19 ICUs and 1/7 in both COVID-19 and non-COVID-19 ICUs), one study each was performed on non-ICU patients, on COVID-19 patients and on general hospitalised patients (all hospital units). The overall percentage (range) of patients with candidaemia among COVID-19 ICU patients was 15% (3–39%). Individual studies found lower proportions among non-COVID-19 non-ICU patients (0.2%), COVID-19 patients (0.4%) and COVID-19 non-ICU patients (0.6%) as opposed to non-COVID-19 ICU patients (27%). Over the year range of these studies (2020–2022), the most commonly isolated *Candida* spp. was *C. parapsilosis* species complex (SC) (49% of all *Candida* isolates, range: 17–74%), followed by *C. albicans* (30%, range: 0–66%) and *Nakaseomyces glabratus* (formerly known as *C. glabrata*) SC (9%, range: 0–17%). *C. auris* was only detected in three of four hospitals located in Athens (overall isolation rate: 14%, range: 4–17%). Antifungal susceptibility data were available in one of the seven studies, which mentioned the overall resistance rate only for fluconazole (48%).

**Table 1:** Epidemiological aspects of candidaemia in Greece during the COVID-19 era as outlined in various studies presented in chronological order (n = 7)

Author [ref.]	Observation time period	Study design (geographic region)	Study population	Number of cases	Candidaemia cases		*Candida* spp.		Susceptibility testing: method (interpretation protocol); % R isolates	Comment
					Number of patients	%	Species	%		
Ioannou et al. [36]	Mar 2020–Aug 2022	Retrospective, observational, single-centre (Crete)	Adult COVID-19 patients	6	6/1,536 patients	0.4	*Candida albicans*	66	NA	The antifungal susceptibility data of bloodstream isolates are not reported separately
							*Candida parapsilosis* SS	17		
							*Nakaseomyces glabratus*	17		
Boattini et al. [27]	Jan 2020–Dec 2021	Retrospective, observational, single-centre (Ioannina)	General patient population (age not reported)	NA						Multicentre surveillance in six southern European hospitals, the epidemiological data of each participating centre are not reported separately *C. auris* was not detected
Giannitsioti et al. [5]	Sep 2020–Oct 2021	Prospective, observational, single-centre (Athens)	Adult non-ICU patients	49	COVID-19		COVID-19		NA	NA
							*C. parapsilosis* SC	45		
					11/1,985 patients	0.6	*C. albicans*	45		
							Other spp.	10		
					non-COVID-19		non-COVID-19			
					38/19,973 patients	0.2	*C. albicans*	45		
							*C. parapsilosis* SC	32		
							*N. glabratus* SC	8		
							*Candida auris*	5		
							*Candida tropicalis*	3		
							Other spp.	7		
Routsi et al. [8]	Mar 2020–Oct 2021	Retrospective, observational, single-centre (Athens)	Adult COVID-19 ICU patients	62	62/600 patients	10	*C. parapsilosis* SS	50	Vitek 2 (EUCAST breakpoint tables v 10.0); 48% FLC-R isolates	Significant increase of incidence (4% during 2005–2008 and 2012–2015) Gradual decrease in the isolation rate of *C. albicans* (39% in 2005–2008, 38% in 2012–2015), accompanied by the emergence of *C. auris* Increase in overall FLC resistance (32% in 2005–2008, 38% in 2012–2015)
							*C. albicans*	24		
							*C. auris*	15		
							*N. glabratus*	9		
							*C. tropicalis*	2		
Papadimitriou-Olivgeris et al. [13]	Apr 2020–Aug 2021	Retrospective, observational, single-centre (Patras)	Adult ICU patients	114	COVID-19		COVID-19		NA	Significant increase of incidence: 5.2/100 unit admissions (4.5/1,000 patient days) in the pre-pandemic era vs 33.6/100 unit admissions (16.9/1,000 patient days) in the COVID-19 era Significant rise in the isolation rate of non-*albicans Candida* spp. only in the non-COVID-19 group (71% in the pre-pandemic era vs 92% in the COVID-19 era)
					76/196 patients (38.8/100 unit admissions, 19.1/1,000 patient days)	39	*C. parapsilosis* SC	50		
							*C. albicans*	32		
							*N. glabratus* SC	7		
							*C. tropicalis*	7		
							Other spp.	4		
					non-COVID-19		non-COVID-19			
					38/143 patients (26.6/100 unit admissions, 13.8/1,000 patient days)	27	*C. parapsilosis* SC	74		
							*N. glabratus* SC	13		
							*C. albicans*	8		
							*C. tropicalis*	5		
Koukaki et al. [7]	Aug 2020–Nov 2020	Retrospective, observational, single-centre (Athens)	Adult COVID-19 ICU patients	6	6/178 patients	3	*C. parapsilosis* SC	17	NA	NA
							*C. auris*	17		
							*N. glabratus* SC	17		
							Other spp.	49		
Kokkoris et al. [14]	Mar 2020–May 2020	Retrospective, observational, single-centre (Athens)	Adult COVID-19 ICU patients	7	7/50 patients	14	*C. albicans*	57	NA	NA
							*C. parapsilosis* SC	43		

EUCAST: European Committee on Antimicrobial Susceptibility Testing; FLC: fluconazole; ICU: intensive care unit; NA: not available; R: resistant; SC: species complex; SS: sensu stricto.

### Demographic data

During the COVID-19 era (2020–2023), 200,753 adults were hospitalised in Attikon. Overall, 365 candidaemic patients were recorded, of whom five (1%) had separate episodes with different *Candida* spp. during hospitalisation. In particular, 154 cases (157 episodes), 89 (58%) male and 65 (42%) female with a median age (range) of 69 (28–97) years, were identified during the early phase of the pandemic (2020-2021). At the onset of the infection, 61/154 (40%) had COVID-19, while the crude within hospital mortality rate was 61% (94/154 patients). During the late COVID-19 era (2022–2023), 211 cases (213 episodes), 132 (63%) male and 79 (37%) female with a median age (range) of 70 (18–97) years, were identified. At the onset of *Candida* BSI, 55/211 (26%) had COVID-19, whereas the crude mortality rate during hospitalisation was 68% (144/154 patients) (Table 2).

**Table 2:** Demographic and clinical characteristics of candidaemic patients at Attikon University General Hospital of Athens, Greece, 2020–2023

Patients’ characteristics	Early COVID-19 era ^a^ (n = 154)		Late COVID-19 era ^b^ (n = 211)		Total (n = 365)	
	n	%	n	%	n	%
Sex						
Male	89	58	132	63	221	61
Female	65	42	79	37	144	39
Median age in years (range)	69 (28–97)		70 (18–94)		70 (18–97)	
Underlying disease						
Haematologic/solid organ malignancy	21	14	35	17	56	15
COVID-19	58	38	47	22	105	29
Haematologic/solid organ malignancy and COVID-19	3	2	8	4	11	3
Other diseases/conditions	72	46	121	57	193	53
Crude within hospital mortality	94	61	144	68	238	65
Patients with haematologic/solid organ malignancy	12	13	22	15	34	14
Patients with COVID-19	34	36	38	26	72	30
Patients with haematologic/solid organ malignancy and COVID-19	2	2	4	3	6	3
Other patients	46	49	80	56	126	53

^a^ Between 1 January 2020 and 31 December 2021.

^b^ Between 1 January 2022 and 31 December 2023.

### Incidence of candidaemia

**Figure 1:**
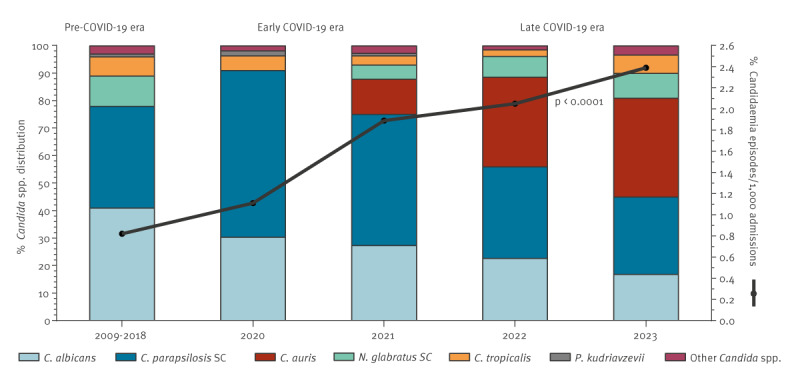
Species distribution of *Candida* bloodstream isolates and changes in candidaemic episodes (per 1,000 hospital admissions) before the COVID-19 pandemic (2009–2018) [15], and during the early (2020–2021) and late (2022–2023) pandemic era, Attikon University General Hospital of Athens, Greece

The overall incidence of candidaemia increased significantly from 0.8 (range: 0.7–0.9) episodes per 1,000 hospital admissions in the pre-COVID-19 era to 1.5 (range: 1.1–1.9) episodes per 1,000 hospital admissions in the early COVID-19 era and further to 2.2 (range: 2.0–2.4) episodes per 1,000 hospital admissions in the late COVID-19 era (p < 0.0001). The respective values for episodes by 10,000 hospital bed days were 2.0 (range: 1.6–2.3), 3.9 (range: 2.7–4.9) and 5.1 (range: 4.6–5.4); p < 0.0001) (Figure 1). In total, almost half (154/370, 42%) of the episodes occurred in ICUs, of which 80/154 (52%) were in COVID-19 ICUs.

Statistically significant differences between the isolation rates of *C. albicans*, *C. parapsilosis* SC and *C. auris* as well as in the incidence of candidaemia were recorded over the years from the pre-COVID-19 era to the end of the late COVID-19 era.

#### Intensive care unit setting

The overall incidence of *Candida* BSI in the ICU setting was 57.2 (range: 28.8–72.0) episodes per 1,000 ICU admissions in the early COVID-19 era and 41.3 (28.2–54.8) episodes per 1,000 ICU admissions in the late COVID-19 era (values for episodes/10,000 ICU bed days were: 39.0, range: 19.1–49.6 and 36.4, range: 28.4–42.7). This shows a significant increase compared with the pre-COVID-19 years 2009–2018 (17.5, range: 2.5–32.2 episodes per 1,000 ICU admissions, p = 0.019 and 9.9, range: 1.1–21.5 episodes/10,000 ICU bed days, p = 0.003).

The incidence of candidaemia in COVID-19 and non-COVID-19 ICU patients was 94.9 (range: 50.6–104.3) episodes per 1,000 COVID-19 ICU admissions vs 35.1 (range: 23.7–44.0) episodes per 1,000 non-COVID-19 ICU admissions (values for episodes/10,000 ICU bed days were: 51.9, range: 30.9–55.8 and 27.9, range: 16.1–40.4) during the early COVID-19 era, and 95.6 (range: 85.1–155.2) episodes per 1,000 COVID-19 ICU admissions vs 28.6 (range 20.6–40.1) episodes per 1,000 non-COVID-19 ICU admissions during the late COVID-19 era for (values for episodes/10,000 ICU bed days were: 61.0, range: 55.7–86.8 and 27.6, range: 21.8–34.2) (Supplementary Figure S1).

#### Non-intensive care unit setting

As regards the non-ICU setting, the overall incidence of candidaemia increased significantly from 0.7 (range: 0.6–0.8) episodes per 1,000 hospital non-ICU admissions in the pre-COVID-19 era to 0.9 (range: 0.9–0.9) episodes per 1,000 hospital non-ICU admissions in the early COVID-19 era and further to 1.3 (range: 1.2–1.6) episodes per 1,000 hospital non-ICU admissions in the late COVID-19 era (p < 0.0001). The values for episodes per 10,000 hospital non-ICU bed days were: 1.8, range: 1.3–2.2; 2.2, range 2.2–2.3 and 3.3, range: 3.2–3.3 (p < 0.0001) (Supplementary Figure S1).

Corresponding comparisons between COVID-19 and non-COVID-19 patients hospitalised in internal medicine and surgical wards could not be conducted since several of them were converted into non-critical care isolation units exclusively assigned to treat COVID‐19 patients for various time periods depending on the gravity of the COVID-19 transmission waves in Greece. Thus, the exact COVID-19 hospitalisation numbers in the non-ICU setting were not available. Only one of the 37 (3%) candidaemic patients who were hospitalised at specialised COVID-19 wards, had previously been admitted to a COVID-19 ICU.

### *Candida* spp. distribution

Among the 370 candidaemia episodes, 23 (6%) were mixed (22 with two *Candida* spp. and one with three *Candida* spp.). The proportion of mixed *Candida* BSI during the early COVID-19 era (16/157, 10%) but not during the late COVID-19 era (7/213, 3%) was significantly higher than that before the pandemic (5%, p = 0.02 and p = 0.53, respectively).

Significant changes in the distribution of *Candida* bloodstream isolates were noted during the COVID-19 era. Overall, 394 *Candida* isolates were recovered, of which 173 (44%) were isolated in the early COVID-19 era. The predominant species causing candidaemia during the COVID-19 era (2020–2023) was *C. parapsilosis* SC (159/394, 40%) similar to pre-COVID-19 era (37%), although its isolation rate declined significantly when the pandemic was nearing its end (90/173, 52% early COVID-19 era; and 69/221, 31% late COVID-19 era, p < 0.0001). Similarly, a considerable decrease over time was recorded for *C. albicans* (41% pre-COVID-19; 49/173, 28% early COVID-19 era; and 47/221, 21% late COVID-19 era, p < 0.0001). On the other hand, *C. auris* emerged as a significant causative agent of candidaemia throughout the COVID-19 era (0% pre-COVID-19; 15/173, 9% early COVID-19 era; and 73/221, 33% late COVID-19 era, p < 0.0001). No significant change was observed in the proportions of other *Candida* spp. *C. auris* was the leading pathogen of *Candida* BSI (30/89 isolates, 34%), followed by *C. parapsilosis* SC (25/89, 28%) and *C. albicans* (17/89, 19%) in 2023 (Figure 1).

#### Intensive care unit setting

Concerning the ICU setting, an analogous shift towards *C. auris* was recorded among the 162 recovered isolates. Namely, *C. parapsilosis* SC (43% pre-COVID-19; 37/75, 49% early COVID-19 era; and 30/87, 34% late COVID-19 era, p = 0.16) and *C. albicans* rates (46% pre-COVID-19; 21/75, 28% early COVID-19 era; and 17/87, 20% late COVID-19 era, p = 0.004) decreased in parallel with a noteworthy increase in *C. auris* rates (0% pre-COVID-19; 10/75, 16% early COVID-19 era; and 34/87, 39% late COVID-19 era, p < 0.0001). No significant change was recorded in the proportions of other *Candida* spp. No difference was noted in the overall dispersion of *Candida* isolates between COVID-19 and non-COVID-19 ICUs (p > 0.10). Worryingly, *C. auris* was the major aetiological agent of ICU-acquired candidaemias (13/29, 45%), followed by *C. parapsilosis* SC (10/29, 34%) and *C. albicans* (4/29, 14%), in 2023 (Supplementary Figure S2).

#### Non-intensive care unit setting

Regarding the non-ICU setting, a corresponding trend in species distribution was observed among the 232 recovered isolates. In particular, *C. parapsilosis* SC (36% pre-COVID-19; 53/98, 54% early COVID-19 era; and 39/134, 29% late COVID-19 era, p = 0.0004) and *C. albicans* rates (41% pre-COVID-19; 28/98, 29% early COVID-19 era; and 30/134, 22% late COVID-19 era, p = 0.0002) decreased significantly, while *C. auris* rates rose substantially (0% pre-COVID-19; 5/98, 5% early COVID-19 era; and 39/134, 29% late COVID-19 era, p < 0.0001). No significant change was noted in the proportions of other *Candida* spp. Notably, *C. auris* was the main causative pathogen of candidaemia outside the ICU setting (17/60, 28%), followed by *C. parapsilosis* SC (15/60, 25%) and *C. albicans* (13/60, 22%), in 2023 (Supplementary Figure S2).

The distribution of *Candida* bloodstream isolates did not differ significantly among COVID-19 patients hospitalised in ICUs or wards (p > 0.11). However, in non-ICU patients, *C. parapsilosis* SC BSI were considerably more common in COVID-19 patients than in non-COVID-19 patients (65% vs 39%, p = 0.006) while the rate of *N. glabratus*-driven candidaemias was significantly lower (0% vs 12%, p = 0.03).

### Antifungal susceptibility profile

Information on in vitro antifungal susceptibility was available for a subset of 336/394 (85%) isolates (Table 3 and Supplementary Table S1). All strains were non-resistant/wild type to amphotericin B and echinocandins except one *C. auris* isolate (pan-echinocandin-resistant harbouring an *FKS1* S639F mutation with anidulafungin, caspofungin and micafungin CLSI MICs > 8 mg/L), which was recovered from a haematologic COVID-19 ICU patient who had experienced repeated exposure to anidulafungin. Among the azoles tested, reduced susceptibility to fluconazole was mostly seen. Namely, 1% of *C. albicans*, 84% of *C. auris* and 32% of *C. parapsilosis* SC strains were fluconazole-resistant, whereas 2% of *C. parapsilosis* SC isolates were categorised as susceptible at increased exposure.

**Table 3:** In vitro susceptibility profile to nine antifungals of major *Candida* spp. bloodstream isolates from patients, Attikon University General Hospital of Athens, Greece, 2020–2023

*Candida* spp. (number of isolates) and antifungal agent	MIC range (mg/L)	MIC_50_ (mg/L)	MIC_90_ (mg/L)	Clinical breakpoints^a^								ECVs/ECOFFs^b^			
				Non-R		S		I		R		WT		non-WT	
				n	%	n	%	n	%	n	%	n	%	n	%
*Candida albicans* (n = 80)															
Anidulafungin	≤ 0.008–0.03	≤ 0.008	0.016	NA		80	100	NA		0	0	ND			
Caspofungin	≤ 0.06–0.5	0.125	0.25	NA											
Micafungin	≤ 0.008–0.016	≤ 0.008	0.016	NA		80	100	NA		0	0	ND			
Isavuconazole	≤ 0.008–0.016	≤ 0.008	≤ 0.008	NA											
Fluconazole	≤ 0.06–64	0.25	0.25	NA		79	99	0	0	1	1	ND			
Itraconazole	≤ 0.016–0.06	≤ 0.016	≤ 0.016	NA		80	100	NA		0	0	ND			
Posaconazole	≤ 0.008–0.03	≤ 0.008	0.016	NA		80	100	NA		0	0	ND			
Voriconazole	≤ 0.008–0.06	≤ 0.008	0.016	NA		80	100	0	0	0	0	ND			
Amphotericin B	≤ 0.125–0.5	0.125	0.25	NA		80	100	NA		0	0	ND			
*Candida auris* (n = 74)															
Anidulafungin	≤ 0.016- > 8	0.03	0.125	73	99	NA		NA		1	1	ND			
Caspofungin	≤ 0.06- > 8	0.5	0.5	73	99	NA		NA		1	1	ND			
Micafungin	≤ 0.016- > 8	0.03	0.06	73	99	NA		NA		1	1	ND			
Isavuconazole	≤ 0.008–0.125	≤ 0.008	0.03	NA								74	100	0	0
Fluconazole	8- > 64	32	> 64	12	16	NA		NA		62	84	ND			
Itraconazole	≤ 0.016–0.5	≤ 0.016	0.125	NA								73	99	1	1
Posaconazole	≤ 0.008–0.125	≤ 0.008	0.03	NA								74	100	0	0
Voriconazole	0.03–2	0.125	1	NA								72	97	2	3
Amphotericin B	0.25–1	0.5	0.5	74	100	NA		NA		0	0	ND			
*Nakaseomyces glabratus* (n = 23)															
Anidulafungin	≤ 0.008–0.06	0.03	0.06	NA		23	100	NA		0	0	ND			
Caspofungin	0.125–0.5	0.5	0.5	NA											
Micafungin	≤ 0.008–0.016	≤ 0.008	0.016	NA		23	100	NA		0	0	ND			
Isavuconazole	0.016–0.25	0.06	0.25	NA											
Fluconazole	2–16	4	16	NA				23	100	0	0	ND			
Itraconazole	0.06–2	0.25	1	NA								23	100	0	0
Posaconazole	0.03–1	0.125	1	NA								23	100	0	0
Voriconazole	0.016–0.5	0.125	0.25	NA								23	100	0	0
Amphotericin B	0.125–0.5	0.125	0.5	NA		23	100	NA		0	0	ND			
*Candida parapsilosis* SS (n = 132)															
Anidulafungin	0.25–4	2	2	NA		132	100	NA		0	0	ND			
Caspofungin	0.5–2	1	1	NA											
Micafungin	0.25–2	1	2	NA		132	100	NA		0	0	ND			
Isavuconazole	≤ 0.008–0.125	0.016	0.03	NA											
Fluconazole	≤ 0.06 – > 64	1	16	NA		87	66	3	2	42	32	ND			
Itraconazole	≤ 0.016–0.25	0.03	0.125	NA		131	99	NA		1	1	ND			
Posaconazole	≤ 0.008–0.125	0.016	0.06	NA		131	99	NA		1	1	ND			
Voriconazole	≤ 0.008–2	0.03	0.25	NA		93	70	27	21	12	9	ND			
Amphotericin B	0.125–1	0.25	0.5	NA		132	100	NA		0	0	ND			
*Candida tropicalis* (n = 14)															
Anidulafungin	≤ 0.008–0.03	0.016	0.03	NA		14	100	NA		0	0	ND			
Caspofungin	0.125–0.5	0.25	0.25	NA											
Micafungin	≤ 0.008–0.03	0.016	0.03	NA								14	100	0	0
Isavuconazole	≤ 0.008–0.03	≤ 0.008	0.03	NA											
Fluconazole	0.25–2	0.5	2	NA		14	100	0	0	0	0	ND			
Itraconazole	≤ 0.016–0.125	0.03	0.125	NA		14	100	NA		0	0	ND			
Posaconazole	0.016–0.06	0.016	0.06	NA		14	100	NA		0	0	ND			
Voriconazole	≤ 0.008–0.125	0.06	0.06	NA		14	100	0	0	0	0	ND			
Amphotericin B	0.125–0.5	0.25	0.5	NA		14	100	NA		0	0	ND			

ECVs/ECOFFs: epidemiological cut-off values; I: intermediate; MIC: minimum inhibitory concentration; NA: not applicable; ND: not determined; Non-R: non-resistant; R: resistant; S: susceptible; SS: sensu stricto; WT: wild type.

^a^ Centers for Disease Control and Prevention’s tentative resistance breakpoints for *C. auris* [17] and European Committee on Antimicrobial Susceptibility Testing (EUCAST) clinical breakpoints for the other *Candida* spp. were used (where available).

^b^ Clinical and Laboratory Standards Institute ECVs for *C. auris* [18] and EUCAST ECOFFs for the other *Candida* spp. were used (where available).

**Figure 2:**
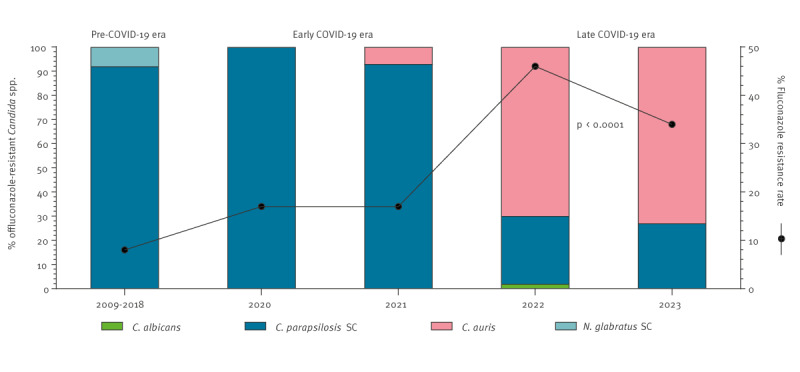
Trends of fluconazole-resistant *Candida* spp. isolates before the COVID-19 pandemic (2009–2018) [15], and in patients during the early (2020–2021) and late (2022–2023) pandemic era, Attikon University General Hospital of Athens, Greece

The fluconazole-resistant *C. parapsilosis* SC strains were isolated from both COVID-19 patients (43%) and non-COVID-19 patients (57%) and were found in different units (36% in COVID-19 ICUs, 27% in non-COVID-19 ICUs, 7% in COVID-19 wards and 30% in non-COVID-19 wards). In addition, those with the highest MICs for fluconazole (≥ 32 mg/L) were also voriconazole-resistant (83%). Their isolation rate was steadily rising from 20% during the pre-pandemic era to 24% in the early COVID-19 era and further to 33% in the late COVID-19 era (p = 0.06). Overall, the recovery frequency of fluconazole-resistant isolates, regardless of species, increased significantly from 8% in the pre-pandemic era to 17% in the early and 41% in the late COVID-19 eras (p < 0.0001) (Figure 2).

Statistically significant increase was observed over the years studied.

### Antifungal consumption

**Figure 3:**
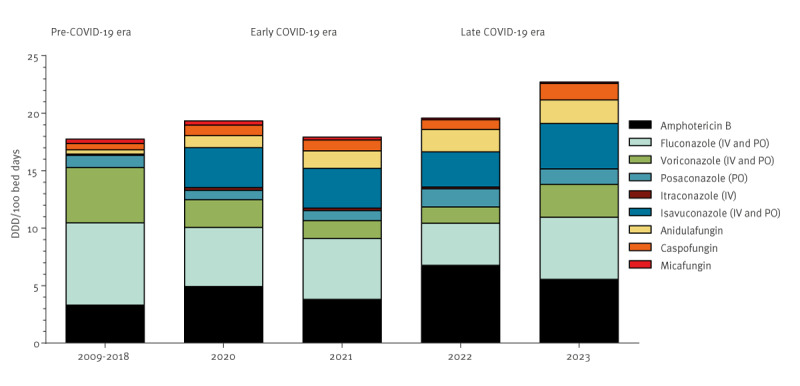
Consumption trend of antifungal agents before the COVID-19 pandemic (2009–2018) [15], and in patients during the early (2020–2021) and late (2022–2023) pandemic era, Attikon University General Hospital of Athens, Greece

DDD: defined daily doses; IV: intravenous; PO: oral.

The overall consumption of antifungal drugs remained stable over the years during the pre-COVID-19 and COVID-19 eras, with a mean of 17.9 (range: 12.8–24.7), 18.7 (range: 18.0–19.4) and 21.2 (range: 19.6–22.8) DDD per 100 hospital bed days during the pre-COVID-19, early COVID-19 and late COVID-19 era, respectively (p = 0.50). In particular, the consumption of voriconazole diminished significantly (p = 0.03), while a significant increase in the consumption of amphotericin B, anidulafungin and caspofungin (p < 0.01) was observed. The consumption of fluconazole, posaconazole, itraconazole, isavuconazole and micafungin remained stable (p > 0.16) (Figure 3).

## Discussion

This study describes the epidemiological trends of *Candida* BSI in the patient population at Attikon University General Hospital of Athens, Greece in the wake of the COVID-19 pandemic. During the COVID-19 era (2020-2023), a significant rise in the incidence of candidaemia was observed in both ICU and non-ICU settings with most candidaemic patients admitted for reasons other than COVID-19. A shift from *C. albicans* to *C. parapsilosis* SC as the main aetiological agent of candidaemia was detected, along with an increased resistance rate to fluconazole for *C. parapsilosis* SC. Worryingly, *C. auris*-driven BSI and their transmission dynamics in the hospital environment have emerged in the transition to the post-pandemic era, raising the overall rate of fluconazole-resistant *Candida* BSI isolates to 41% at the end of the COVID-19 era in 2023.

The constantly evolving epidemiology of candidaemia globally has significant implications for patient management and highlights the need for continuous regional monitoring [12], specifically during extreme events such as the unexpected COVID-19 pandemic, which may have a considerable impact on the epidemiological pattern of healthcare-associated infections [2]. To date, the contemporary prevalence and species distribution of candidaemia in Greece remains largely unknown and data on the antifungal resistance profile of the involved bloodstream isolates are lacking. During the COVID-19 era, the majority of studies on *Candida* BSI in Greece were carried out in a COVID-19 ICU setting during the early phase of the pandemic and included few patients. As a consequence, their results may not be broadly applicable to all candidaemic patients reflecting the current at that time local spectrum of the infection. Therefore, we aimed to provide an overview of the epidemiology of *Candida* BSI in the patient population at Attikon University General Hospital of Athens, encompassing the beginning of the pandemic and the WHO’s declaration of the end of the COVID-19 emergency phase.

The incidence rate of candidaemia at Attikon has been stable over a 10-year period (2009–2018) [15]. Nevertheless, a nearly threefold increase was noted during the pandemic. In total, we identified a higher number of candidaemic episodes in patients admitted to Attikon for reasons other than COVID-19 (249/365, 68%), as previously described (64–74%) [20-22]. Two- to threefold differences have been also recorded in single-centre studies that compared the incidence of *Candida* BSI before and during the COVID-19 pandemic in Spain (1.4 vs 2.6 cases/10,000 patient days) [20] and Brazil (0.2 vs 0.6 cases/1,000 patient days) [22], while no data have been previously reported for Greek hospitals precluding country-wide comparisons. Such an increase could be potentially attributed to either the expansion of the number of patients prone to develop candidaemia [1] or the higher propensity for patient-to-patient transmission since the COVID-19 pandemic negatively impacted infection control measures.

The difference was more pronounced in the context of the ICU setting. The overall incidence of *Candida* BSI rose considerably (almost a fourfold increase in bed days) during the pandemic, with the infection rate being higher in COVID-19 patients, as previously described [20]. Of note, several retrospective studies have shown a significantly increased incidence of candidaemia among COVID-19 patients requiring ICU care in comparison to historical non-COVID-19 cohorts [23,24], which is in agreement with our findings. Nevertheless, these data should be interpreted with caution given that the patient populations being compared are different in terms of pathology and underlying comorbidities. Moreover, our results are consistent with a previously published comparator study reporting that the incidence of *Candida* BSI increased also in the non-COVID-19 group during the pandemic [22]. The difference observed may have been driven by the fact that hospital admissions for non-COVID-19 cases were restricted to severe aetiologies, i.e. patients who were more likely to have longer length of stay and higher risk for nosocomial infections, in conjunction with impaired infection control practices during the surge of the pandemic.

Regarding the local epidemiology, analogous trends have been previously recorded across two Greek adult ICUs. A single-centre study performed in Athens found an almost threefold increased incidence rate of candidaemia in COVID-19 patients as compared with historical non-COVID-19 controls (103.3 vs 38.0–42.0/1,000 ICU admissions) [8], which is lower than the fivefold increase observed at Attikon Hospital. On the other hand, a single-centre study carried out in Patras (south-western Greece) reported a nearly fourfold greater incidence density of ICU-acquired candidaemia during the pandemic era (4.5 vs 16.9/1,000 patient days), in line with our findings. Nevertheless, similar rates were recorded among COVID-19 and non-COVID-19 patients in the same time period (19.1 and 13.8/1,000 patient days, respectively) [13], as opposed to our study. Similar intra-country variations have been previously described [25], probably due to differences in the time period of the pandemic regarding the impact of its diverse transmission waves, local practices and surveillance protocols, the consumption of antifungal drugs and the patient populations taking into account the variable percentages of surgical/oncological cases.

Amid the pandemic, *C. parapsilosis* SC outranked *C. albicans* as the dominant causative agent of candidaemia at Attikon Hospital (40% vs 37% in the pre-COVID-19 era) [15]. This is in accordance with data derived from southern European countries (Italy and Spain) demonstrating a corresponding shift in favour of *C. parapsilosis* SC-driven BSI in the general patient population during the COVID-19 era [25,26]. Overall, the proportion of other previously isolated non-*albicans Candida* spp. such as *C. tropicalis*, which increased in Brazil [22], the US [21] and Spain [25] in the same time period, remained stable at Attikon. No difference was observed in the dispersion of *Candida* bloodstream isolates between COVID-19 and non-COVID-19 ICUs during the pandemic, which is in line with worldwide trends [23,24]. However, significant differences in species distribution outside of the ICU setting were noted, like the dominance of *N. glabratus* candidaemic episodes in non-COVID-19 patients, as previously described [27]. A possible explanation would be that COVID-19 patients do not have the risk factors commonly linked to the development of *N. glabratus* invasive infections, particularly recent abdominal surgery and exposure to azoles.

Yet, our most concerning finding was the dissemination of *C. auris* candidaemias altering the in-hospital spectrum of the infection. The spread of this nosocomial pathogen was probably aggravated by the COVID-19 pandemic [2]. Overall, 89 *C. auris*-driven BSI were recorded between March 2021 and December 2023, and the species predominance (34%) in 2023 is worrisome. All *C. auris* isolates clustered in clade I and displayed an identical short tandem repeat genotype (data not shown). Most of them (84%) were fluconazole-resistant and had elevated MICs to the other azoles compared with the corresponding MICs of the primary *C. auris* isolated in Japan in 2009 [28]. Despite the fact that some isolates had fluconazole MICs lower than the CDC’s tentative resistance breakpoint of 32 mg/L [17], the use of azoles against those isolates is questionable since breakthrough *C. auris* infections (i.e. infections occurring in patients receiving systemic antifungal therapy) caused by fluconazole-non-resistant isolates (MICs 2–8 mg/L) have been reported [29]. Furthermore, we identified a pan-echinocandin-resistant isolate (*FKS1* S639F mutant) that evolved in vivo upon repeated exposure to anidulafungin, prompting for continuous monitoring of *C. auris* antifungal susceptibility patterns.

Another critical issue is that our data illustrated a progressive rise of BSI attributed to fluconazole-resistant *C. parapsilosis* SC regardless of the hospitalisation unit. This is of particular importance since the European guidelines have endorsed fluconazole as the first-line therapy for *C. parapsilosis* SC BSI coupled with prompt catheter removal [10], whereas the systemic use of fluconazole still holds a significant place in the Greek hospital sector. This is also shown in the present study where fluconazole was one of the most commonly prescribed antifungal drugs. In contrast to our findings, Routsi et al. [8] observed that fluconazole resistance in *C. parapsilosis sensu stricto* (SS) decreased from 56% in 2012–2015 to 49% in 2020–2021 in a Greek ICU that served only COVID-19 patients during the pandemic. However, the overall isolation rate of fluconazole-resistant isolates regardless of species increased over time (32% in 2005–2008, 38% in 2012–2015 and 48% in 2020–2021), which is in line with our results [8]. Notably, fluconazole resistance in *C. parapsilosis* SC has emerged rapidly worldwide since 2018, with centres reporting rates greater than 10% sharply increased during the COVID-19 era [30], corroborating our findings during pre-pandemic area published in 2020 [15] and during pandemic era described in the present study. Indicatively, Franconi et al. reported that the rate of fluconazole-resistant *C. parapsilosis* SS candidaemias increased significantly from 7% in 2015 to 63% in 2022 (with 85% of isolates being cross-resistant to voriconazole) in an Italian tertiary academic hospital [26], and a recent multicentre study conducted in the Madrid metropolitan area, Spain, showed that the spread of fluconazole resistance in *C. parapsilosis* SC bloodstream isolates has gained traction during the pandemic (from 3% in 2019 to 37% in 2022) [31]. Globally, when pre-COVID-19 (2018–2019) and COVID-19 (2020) datasets were compared in the context of the SENTRY antifungal surveillance programme, an elevated fluconazole resistance level (from 10% to 14%) was recorded for *C. parapsilosis* SC [32]. It can be speculated that this phenomenon was amplified by the conditions created during the pandemic emergency, including patient overcrowding, deficiency in care-related hygiene and relaxation of basic infection control measures, among others [2]. In fact, most *C. parapsilosis* SS isolates recovered during the COVID-19 era were clonal and most of the fluconazole-resistant ones harboured the Y132F mutation in the *ERG11* gene (data not shown). Moreover, our data suggest that the increase in the incidence of fluconazole-resistant *C. parapsilosis* SC during the COVID-19 era has not been driven by the selective pressure of fluconazole and voriconazole consumption, as previously described [33]. The latter advocates that the main reason for the epidemiological changes described here might have been because of poor adherence of infection prevention and control measures. During the pandemic the following factors that could have contributed to the increase have been noticed: (i) extended wear/reuse of gloves by healthcare workers for the care of multiple patients, (ii) staff movements across different hospital units, (iii) previous hospitalisation in hospitals located in the Attica region where *C. auris* and *C. parapsilosis* outbreaks have been documented [34,35], (iv) systematic screening not being performed for all patients at admission and randomly during hospitalisation, (v) isolation/cohorting of patients colonised with *C. auris* until discharge not implemented due to space and nursing staff shortages.

It should be acknowledged that our study is limited by its retrospective nature, while its results may have been affected by local hospital-associated practices in a period of heavy workload and distinct patient populations. However, it contributes to the existing knowledge regarding the epidemiology of candidaemia in Greece, and particularly the extent of *C. auris*-driven infections, by providing up-to-date data. Hence, the findings presented herein may be an indicator of an emerging regional problem and can be considered as a point of reference paving the way for future epidemiological surveys. In addition, as similar trends have been reported in other countries, mainly in the south of Europe, the present report describes a situation that may lead to the end of the era of empiric azole therapy against *Candida* infections.

## Conclusion

The incidence of *Candida* BSI increased worryingly during the COVID-19 era. *C. auris* emerged among the major causative agents, advocating the need for ongoing vigilance and strict adherence to infection prevention and control practices in healthcare settings, whereas further studies are required to shed light on its potential regional endemicity. Meanwhile, fluconazole resistance in *C. parapsilosis* SC exhibited a rising trend in our large tertiary hospital, which together with the rise of *C. auris* increase the rate of azole-resistant *Candida* isolates to 41% of all clinical isolates. Prompt implementation of surveillance and antifungal stewardship activities is crucial to contain the selection and spread of echinocandin resistance in *C. auris*.

## Supplementary Data

414000 Supplement
