# Supplementary material for: Increase in candidemia cases and emergence of fluconazole-resistant Candida parapsilosis and C. auris isolates in a tertiary care academic hospital during the COVID-19 pandemic, Greece, 2020 to 2023
Source: Euro Surveill. 2024 Jul 18;29(29):2300661. doi: 10.2807/1560-7917.ES.2024.29.29.2300661 (PMC11258949; doi:10.2807/1560-7917.ES.2024.29.29.2300661)
Supplement: Supplement [file 23-00661_MELETIADIS_Supplement.pdf]

This supplementary material is hosted by *Eurosurveillance* as supporting information alongside the article [Increase in candidemia cases and emergence of fluconazole-resistant *Candida parapsilosis* and *C. auris* isolates in a tertiary care academic hospital during the COVID-19 pandemic, Greece, 2020 to 2023], on behalf of the authors, who remain responsible for the accuracy and appropriateness of the content. The same standards for ethics, copyright, attributions and permissions as for the article apply. Supplements are not edited by *Eurosurveillance* and the journal is not responsible for the maintenance of any links or email addresses provided therein.

Table S1. *In vitro* susceptibility profile to nine antifungals of rare *Candida* spp. bloodstream isolates from patients, Attikon University General Hospital of Athens, Greece, 2020-2023

Candida spp. (number of isolates) and antifungal agent	MIC range (mg/L)	MIC_{50} (mg/L)	MIC_{90} (mg/L)	Clinical breakpoints[a]						ECOFFs[b]			
				S		I		R		WT		non-WT	
				n	%	n	%	n	%	n	%	n	%
Candida dubliniensis (n = 1)													
Anidulafungin	0.016	NA	NA	NA									
Caspofungin	0.125	NA	NA	NA									
Micafungin	≤ 0.008	NA	NA	NA									
Isavuconazole	≤ 0.008	NA	NA	NA									
Fluconazole	0.25	NA	NA	1	100	0	0	0	0	ND			
Itraconazole	≤ 0.016	NA	NA	1	100	NA		0	0	ND			
Posaconazole	≤ 0.008	NA	NA	1	100	NA		0	0	ND			
Voriconazole	≤ 0.008	NA	NA	1	100	0	0	0	0	ND			
Amphotericin B	≤ 0.125	NA	NA	1	100	NA		0	0	ND			
Candida metapsilosis (n = 1)													
Anidulafungin	0.25	NA	NA	1	100	NA		0	0	ND			
Caspofungin	0.5	NA	NA	NA									
Micafungin	0.25	NA	NA	1	100	NA		0	0	ND			
Isavuconazole	0.016	NA	NA	NA									
Fluconazole	2	NA	NA	1	100	0	0	0	0	ND			
Itraconazole	0.06	NA	NA	1	100	NA		0	0	ND			
Posaconazole	0.016	NA	NA	1	100	NA		0	0	ND			
Voriconazole	0.06	NA	NA	1	100	0	0	0	0	ND			
Amphotericin B	0.25	NA	NA	1	100	NA		0	0	ND			
Candida orthopsilosis (n = 3)													
Anidulafungin	0.5–1	NA	NA	3	100	NA		0	0	ND			
Caspofungin	1–1	NA	NA	NA									
Micafungin	0.25–1	NA	NA	3	100	NA		0	0	ND			
Isavuconazole	0.016–0.5	NA	NA	NA									
Fluconazole	2– > 64	NA	NA	1	33	0	0	2	67	ND			
Itraconazole	0.03–0.125	NA	NA	3	100	NA		0	0	ND			
Posaconazole	0.03–0.03	NA	NA	3	100	NA		0	0	ND			
Voriconazole	0.03–8	NA	NA	1	33	0	0	2	67	ND			
Amphotericin B	0.125–0.5	NA	NA	3	100	NA		0	0	ND			
Clavispora lusitaniae (n = 4)													
Anidulafungin	0.016–0.125	NA	NA	NA									
Caspofungin	0.5–1	NA	NA	NA									
Micafungin	0.03–0.25	NA	NA	NA									

Isavuconazole	≤ 0.008– ≤ 0.008	NA	NA	NA								
Fluconazole	0.125–0.5	NA	NA	NA								
Itraconazole	≤ 0.016–0.03	NA	NA	NA					4	100	0	0
Posaconazole	≤ 0.008–0.016	NA	NA	NA								
Voriconazole	≤ 0.008–0.016	NA	NA	NA								
Amphotericin B	0.125–0.25	NA	NA	NA					4	100	0	0
Kluyveromyces marxianus (n = 1)												
Anidulafungin	0.016	NA	NA	NA								
Caspofungin	0.25	NA	NA	NA								
Micafungin	0.03	NA	NA	NA								
Isavuconazole	≤ 0.008	NA	NA	NA								
Fluconazole	0.5	NA	NA	NA					1	100	0	0
Itraconazole	0.03	NA	NA	NA								
Posaconazole	0.016	NA	NA	NA								
Voriconazole	≤ 0.008	NA	NA	NA								
Amphotericin B	0.25	NA	NA	NA					1	100	0	0
Meyerozyma guilliermondii (n = 1)												
Anidulafungin	1	NA	NA	NA								
Caspofungin	1	NA	NA	NA								
Micafungin	0.25	NA	NA	NA								
Isavuconazole	0.25	NA	NA	NA								
Fluconazole	16	NA	NA	NA					1	100	0	0
Itraconazole	0.5	NA	NA	NA					1	100	0	0
Posaconazole	0.25	NA	NA	NA					1	100	0	0
Voriconazole	0.25	NA	NA	NA								
Amphotericin B	0.125	NA	NA	NA					1	100	0	0
Pichia kudriavzevii (n = 2)												
Anidulafungin	0.03–0.06	NA	NA	2	100	NA	0	0	ND			
Caspofungin	0.5–1	NA	NA	NA								
Micafungin	0.06–0.125	NA	NA	NA					2	100	0	0
Isavuconazole	≤ 0.008–0.25	NA	NA	NA								
Fluconazole	32–64	NA	NA	NA					2	100	0	0
Itraconazole	0.03–0.25	NA	NA	NA					2	100	0	0
Posaconazole	0.06–0.125	NA	NA	NA					2	100	0	0
Voriconazole	0.25–0.5	NA	NA	NA					2	100	0	0
Amphotericin B	0.25–1	NA	NA	2	100	NA	0	0	ND			

ECOFFs: epidemiological cut-off values; MIC: minimum inhibitory concentration; NA: not applicable; ND: not determined; I: intermediate; R: resistant; S: susceptible; WT: wild type

[a] European Committee on Antimicrobial Susceptibility Testing (EUCAST) clinical breakpoints and ECOFFs were used (where available)

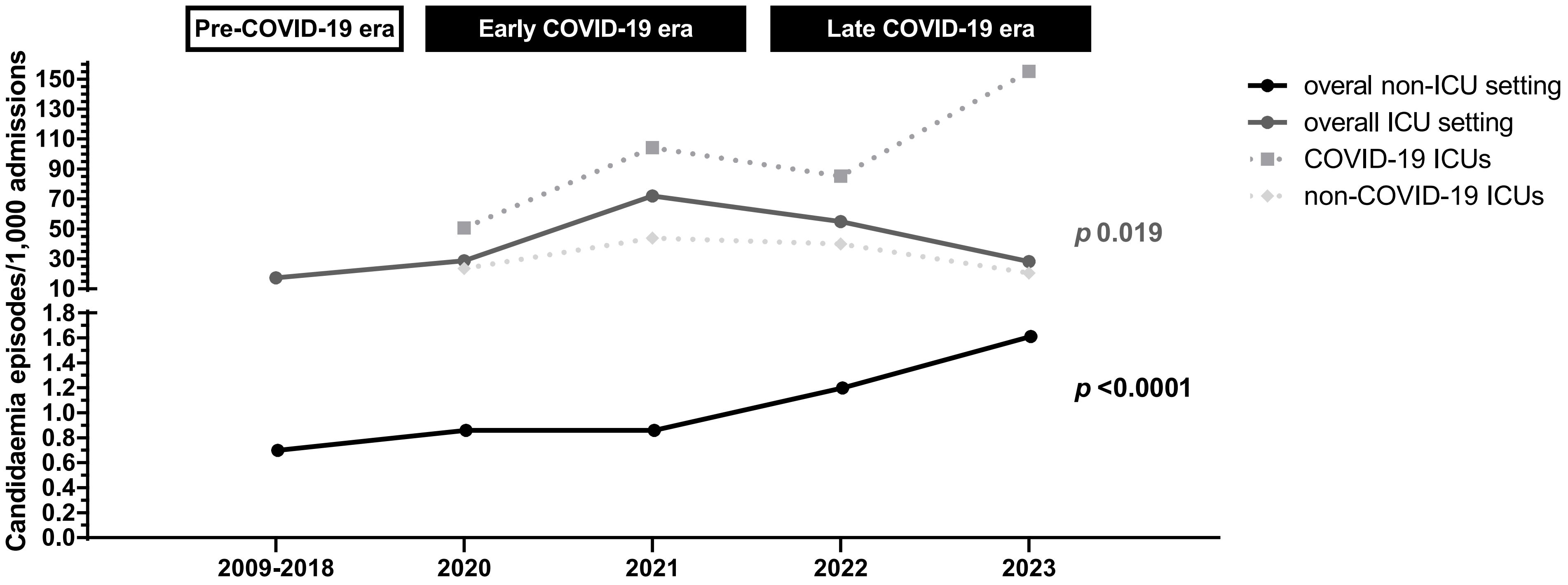

Figure S1. Temporal changes in candidaemic episodes (per 1,000 admissions) in the non-ICU and ICU environments before (2009-2018) (15) and during the COVID-19 pandemic (early [2020-2021] and late [2022-2023] phase). Statistically significant differences in the incidence of candidaemia were recorded over the years to both settings.

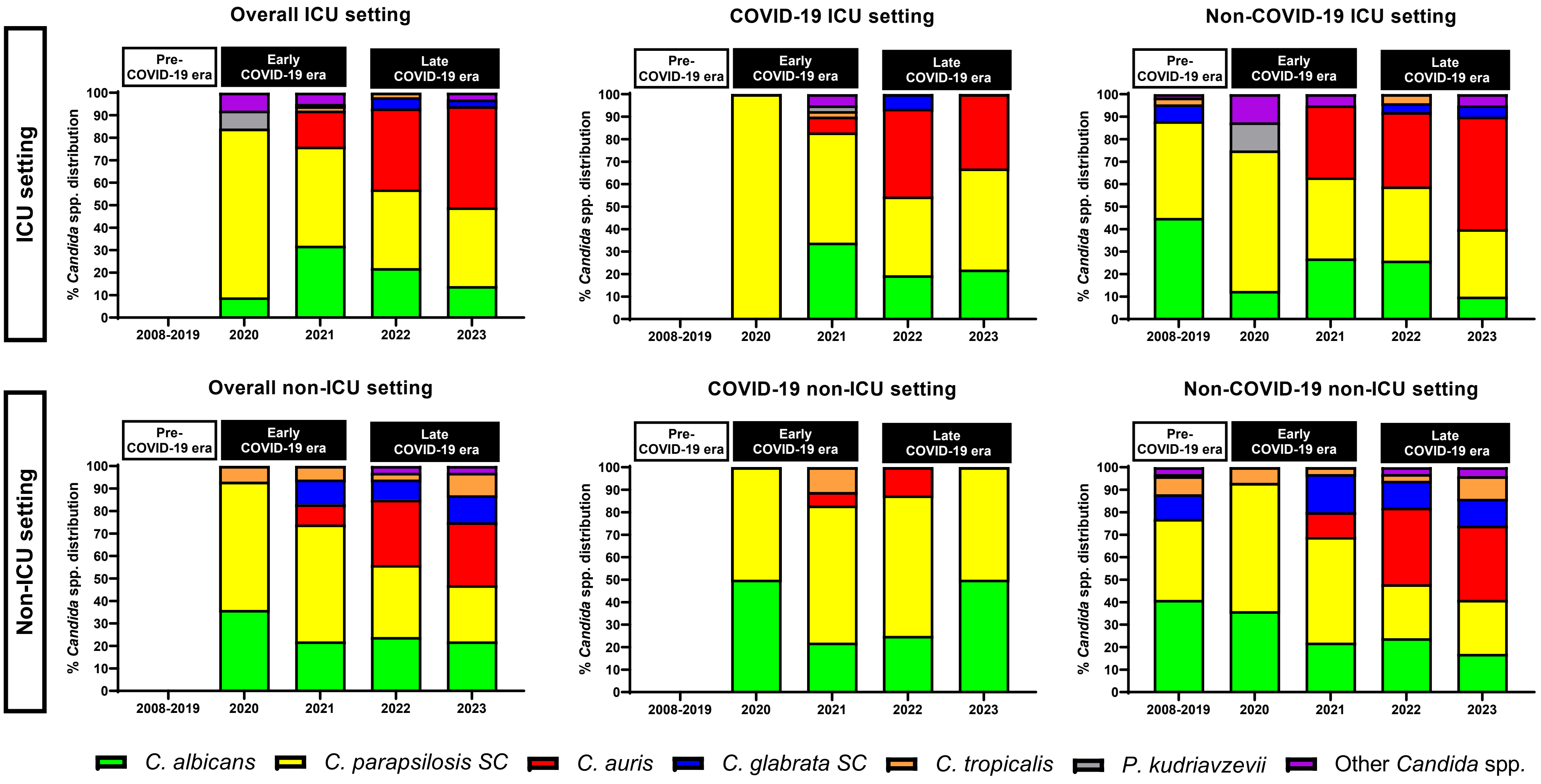

Figure S2. Temporal distributions of *Candida* spp. in the ICU (upper graph) and non-ICU (lower graph) environments before (2009-2018) (15) and during the COVID-19 pandemic (early [2020-2021] and late [2022-2023] phase). The rates of *C. albicans* and *C. parapsilosis* SC decreased in parallel with a substantial increase in *C. auris* rates over the years. No significant change was noted in the proportions of other *Candida* spp.
